# *The Organon* and Materia Medica Club of the Bay Cities of California

**Published:** 1897-10

**Authors:** 


### *THE ORGANON* AND MATERIA MEDICA CLUB OF THE BAY CITIES OF CALIFORNIA.

The regular semi-monthly meeting of *The Organon* and Materia Medica Club of the Bay Cities was held in San Francisco, July 16th, at the office of Dr. G. H. Martin.

There were present: Drs. J. M. and C. M. Selfridge, Augur, Hohngren, J. W. and F. N. Ward, G. H. and E. F. Martin, and Manning.

The meeting was called to order by the President, Dr. J. M. Selfridge.

The minutes of July 2d were read and approved.

On motion the By-Laws were so amended as to do away with associate members, and place all upon an equal footing.

The resignation of Chas. L. Dwyer was read, and on motion accepted.

This being the annual meeting, election of officers took place, with the following result, viz.: Dr. J. M. Selfridge for President; Dr. G. H. Martin for Vice-President; Dr. Guy E. Manning for Secretary and Treasurer; and Dr. J. W. Ward, Augur, and C. M. Selfridge for Censors.

After the paragraphs pertaining to the "Origin of Disease" were read from Hahnemann's *Chronic Diseases*, Dr. J. M. Selfridge read a paper upon

